# Physical Activity Misinformation on Social Media: Systematic Review

**DOI:** 10.2196/62760

**Published:** 2025-10-08

**Authors:** D David Thomas, Linglin Xu, Brian Yu, Octavio Alanis, John Adamek, Imani Canton, Xuan Lin, Yan Luo, Sean P Mullen

**Affiliations:** 1 Informatics Programs University of Illinois Urbana-Champaign Urbana, IL United States; 2 Department of Health and Kinesiology University of Illinois Urbana-Champaign Urbana, IL United States; 3 Shenyang Sport University Shenyang China; 4 Beckman Institute for Advanced Science and Technology University of Illinois Urbana-Champaign Urbana, IL United States; 5 Center for Social and Behavioral Science University of Illinois Urbana-Champaign Urbana, IL United States

**Keywords:** health misinformation, online misinformation, systematic review, social media, physical activity, exercise, kinesiology, online content, digital health, artificial intelligence, AI

## Abstract

**Background:**

Social media is a prominent way in which health information is spread. The accuracy and credibility of such sources range widely, with misleading statements, misreported results of studies, and a lack of references causing health misinformation to become a growing problem. However, previous research on health misinformation related to topics including vaccines, nutrition, and cancer has excluded physical activity despite it being highly searched for and discussed online.

**Objective:**

This systematic review was designed to synthesize the existing literature focused on physical activity misinformation on social media in accordance with PRISMA (Preferred Reporting Items for Systematic Reviews and Meta-Analyses) 2020 guidelines.

**Methods:**

Keyword searches were conducted in PubMed, the Cochrane Library, Web of Science, and Scopus databases for records published between January 2016 and May 2025. This search strategy yielded 9039 articles. Titles and abstracts were screened by independent reviewers, resulting in 168 (1.86%) articles selected for full-text review. After further review, 33 (19.6%) articles met the inclusion criteria and were used in the final synthesis.

**Results:**

For the 33 studies selected, topics included physical rehabilitation and therapeutic exercise recommendations (n=15, 45%), general physical activity and messaging (n=6, 18%), exercising with a specific condition (n=4, 12%), women’s health (n=3, 9%), weight loss (n=2, 6%), exercise testing (n=1, 3%), “immune boosting exercise” (n=1, 3%), and workplace sitting versus standing guidelines (n=1, 3%). The social media platforms YouTube (n=13, 39%), TikTok (n=7, 21%), Facebook (n=2, 6%), Instagram (n=1, 3%), and Pinterest (n=1, 3%) were studied, whereas other articles (n=9, 27%) analyzed content that had not explicitly been posted to social media but could be shared widely online. In total, 4 (12%) studies reported research that proactively engaged participants, and the remaining 29 (88%) studies analyzed readily available online content, including social media, news articles, websites, and blogs. Furthermore, 27 (82%) studies reported at least 1 measure of misinformation prevalence, whereas 21 (64%) reported a metric of reach, and 6 (18%) studies reported a measure of misinformation spread.

**Conclusions:**

Our findings indicate that research on social media physical activity misinformation spans a diverse array of physical activity topics, with YouTube being the most studied platform due to its widespread use and ease of content evaluation. This review also highlights the prevalence of low-quality information across various platforms and a lack of longitudinal investigations. Our review underscores the need for multifaceted research approaches and suggests several strategies to combat misinformation, including improved messaging, high-quality information dissemination by institutions, detailed debunking efforts, and raising awareness about misinformation. Future research should focus on understanding the spread of physical activity misinformation across platforms and its impact, especially on vulnerable populations.

**Trial Registration:**

PROSPERO CRD42022316101; https://www.crd.york.ac.uk/PROSPERO/view/CRD42022316101

## Introduction

### Background

It is well documented that the internet is a key source of health information for many people across the world [1,2]. Although websites, blogs, and social media can provide valuable information about both general and specific health topics [3,4], they can just as easily present misleading or inaccurate information [5,6], termed misinformation. Misinformation is often shared unintentionally by parties unaware of its falsehood, contrasting with disinformation, which is shared with an awareness of its falsity and potential harm [7]. The dissemination of misinformation can be carried out by various actors, including public figures, professionals, educators, and personal contacts.

Although misinformation spans many domains, the realm of physical activity is notably susceptible. Misinformation regarding the safety and benefits of physical activity, particularly newer trends, such as high-intensity interval training (HIIT), can lead to misconceptions about its safety and potential risks, impacting public health guidance and individual behavior [8]. The ubiquitous use of smartphones and social media exacerbates the speed and reach of such misinformation [9], highlighting the urgency to address it specifically within the broader context of health misinformation.

The issue of online health misinformation has become so significant that in 2021, the US surgeon general issued an advisory guiding stakeholders (from individuals to organizations) on actions to combat misinformation [10]. For researchers taking action, the advisory recommends to (1) strengthen the monitoring of health questions, concerns, and misinformation; (2) assess the impact of health misinformation; (3) prioritize understanding how people are exposed to and affected by misinformation and how this may vary for different subpopulations; and (4) evaluate the effectiveness of strategies and policies to prevent and address health misinformation. This work is well underway, as several systematic reviews have been conducted to identify the range and impact of health information research [4,11,12] as well as methods to correct health misinformation on social media [13].

The wide range of health misinformation topics that have been investigated on social media includes pandemics, nutrition, and cancer [4,11,12]. Findings from the study by Suarez-Lledo and Alvarez-Galvez [12] showed that most health-related misinformation research has focused on vaccines and drugs or smoking, with Twitter having the highest prevalence of misinformation across the health topics and platforms investigated. Melchior and Oliveira [11] found that the prevalence of misinformation in health topics reported by studies ranged from none to 98%. This variance in the prevalence of health misinformation on social media is concerning and indicates why this is a growing field of study. Between 2012 and 2018, an 850% increase in published articles was reported, from 2 to 19 published annually on the topic [12].

However, one area that has received limited attention is physical activity misinformation. Despite the interest in health-related misinformation and the well-established benefits of regular physical activity for health and well-being [14], there is still a significant amount of misinformation surrounding this topic online. This is particularly concerning given the importance of physical activity in preventing and managing chronic conditions, such as obesity, diabetes, and heart disease [15]. Although physical activity is generally viewed as safe, there are established, elevated risks of adverse events caused by increasing physical activity while striving to meet public health guidelines [16] and participating in exercise interventions [17], with musculoskeletal injuries being among the most common adverse events—more so among higher intensity exercises [18]. Ekkekakis et al [8] have brought attention to myriad unfounded claims regarding the safety and tolerance for higher intensity physical activity (specifically, HIIT). Extraordinary claims have been made in reference to HIIT, and it is sometimes ignored that HIIT can contribute to injury and adverse cardiovascular events, among other negative consequences. Therefore, it is crucial to understand the prevalence and impact of physical activity misinformation and develop effective strategies to combat it.

### Objective

The purpose of this systematic review was to explore the current state of research on physical activity misinformation on social media, including its reported prevalence, reach, and spread, and strategies for addressing this issue.

## Methods

### Guidelines and Search Strategy

The protocol for this systematic review was preregistered on PROSPERO (CRD42022316101). The procedure and research findings have also been reported in accordance with the PRISMA (Preferred Reporting Items for Systematic Reviews and Meta-Analyses) guidelines [19], see Multimedia Appendix 1. To review the existing literature of physical activity misinformation on social media, we searched the databases PubMed, the Cochrane Library, Web of Science, and Scopus for records published from January 2016 through May 2025, using keywords related to misinformation, social and online media, information transmission, and physical activity. This time frame was selected because research on social media health misinformation shows little scholarship before 2016 [12]. Furthermore, Google Search trends showed interest in “fake news,” and by proxy, the idea of misinformation increased dramatically between October 2016 and February 2017, coinciding with the 2016 US presidential election [20].

Adapting the search strategy of Wang et al [4], our search was structured around four key concepts to ensure comprehensive coverage: (1) misinformation terms, including synonyms such as “health myths” and “inaccurate information,” to capture various forms of information disorders; (2) social media and online platforms to cover a wide range of digital dissemination channels; (3) information transmission terms to identify studies examining the spread and engagement with content; and (4) physical activity terms, including a broad array of activities and exercise types. Medical Subject Headings (MeSH) were used, where possible, to enhance search sensitivity and precision, as presented in Textbox 1.

**MeSH used in this study.**

((misinformation OR disinformation OR “fake news” OR rumor* OR myth* OR “false claims” OR “inaccurate information” OR “misleading information” OR “unsubstantiated claims” OR “health fraud” OR pseudoscience OR fallacy) OR “Communication”[MeSH] OR “Disinformation”[MeSH]) AND ((online OR internet OR social media OR web OR website OR blog* OR forum* OR Twitter OR X OR Facebook OR Instagram OR TikTok OR Snapchat OR YouTube OR Reddit OR Pinterest OR Google OR “search engine” OR “social network*” OR “online community” OR “digital media”) OR “Social Media”[MeSH] OR “Internet”[MeSH]) AND ((spread OR propagat* OR disseminat* OR circulat* OR communicat* OR diffus* OR broadcast* OR share OR sharing OR viral* OR dissemination OR transmission OR reception OR engagement OR amplification) OR “Information Dissemination”[MeSH]) AND ((“physical activity” OR exercise OR “aerobic exercise” OR “aerobic training” OR “strength training” OR “resistance training” OR fitness OR sport* OR recreation OR “active lifestyle” OR “exercise therapy” OR “physical fitness” OR walking OR running OR swimming OR cycling OR yoga OR “weight lifting”) OR “Exercise”[Mesh] OR “Physical Fitness”[Mesh] OR “Resistance Training”[Mesh])

The search yielded 4424 records from PubMed, 238 records from the Cochrane Library, 517 records from Web of Science, and 4041 records from Scopus. All collected references were uploaded into reference management software Zotero (version 7.0; Corporation for Digital Scholarship) for deduplication and subsequent assessment of eligibility. Of the 9220 records, we identified and removed 184 (2%) duplicate records, leaving 9036 records to be reviewed for eligibility. By hand searching, we identified a further 3 records to screen, bringing the total to 9039 records.

### Screening and Study Selection

The full list of unique articles was downloaded from the reference management software and organized in a spreadsheet (Microsoft Excel version 16.0), which was screened by 2 research team members independently for relevance based on title, abstract, and keywords. The inclusion and exclusion criteria mentioned in Textbox 2 were used.

**Inclusion and exclusion criteria for article selection.**

**Inclusion criteria**

- Misinformation: Articles must relate to misinformation, disinformation, or another information disorder.
- Social media: Potential misinformation must be actually or plausibly shared or consumed through social media (eg, an original social media post or sharing an online news article on social media).
- Physical activity: Only articles related to physical activity and exercise were included.
- Other article restrictions: Articles must concern humans sharing or consuming information about human physical activity. Articles must be peer-reviewed original research. Only articles written in English were included.

**Exclusion criteria**

- Reviews, editorials, and gray literature (eg, government reports, policy papers, and dissertations) were excluded.

Upon screening, 168 articles were considered potentially eligible and were marked for full-text review. After reviewing the full articles, 33 (19.6%) articles were found to satisfy all inclusion criteria.

### Data Extraction and Analysis

Following screening, the 33 eligible studies were analyzed for the following information: (1) physical activity topic; (2) social media platform and media type; (3) study population and design; and (4) quantification of misinformation prevalence, reach, and spread. Our plan was to conduct quantitative and qualitative data analyses on the final sample of 33 studies to identify and summarize common themes and measures of physical activity misinformation. While we initially planned to conduct statistical analysis and formal quality assessment of the included studies, heterogeneity in study designs and outcomes reported made this unfeasible, and substantial modifications to standard quality assessment tools would have compromised their validity.

## Results

### Physical Activity Topics

**Figure 1:**
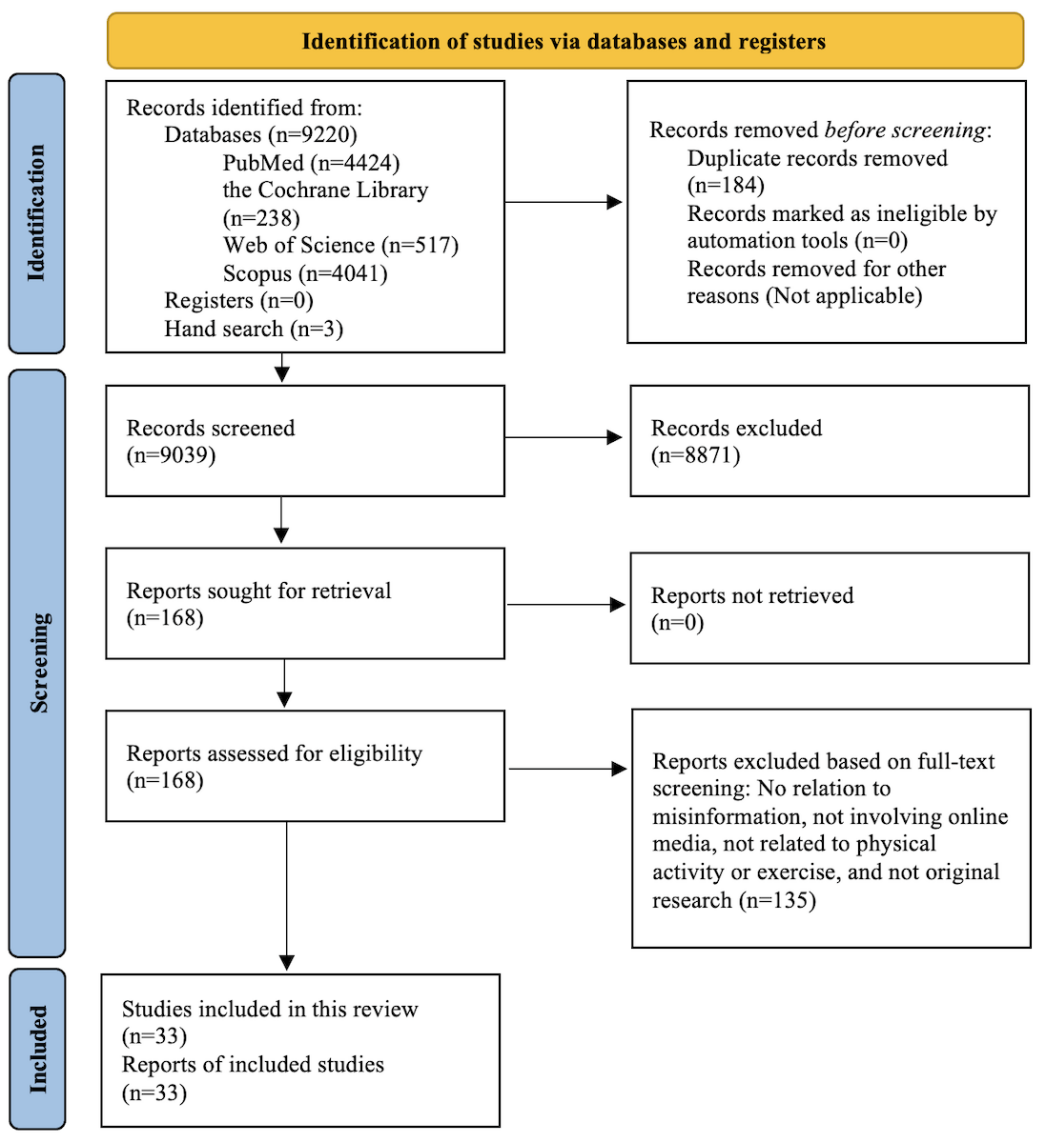
Identification of studies via databases and registers.

In the 33 studies selected during screening (Figure 1), the most common topic was the quality and accuracy of physical rehabilitation and therapeutic exercise recommendations (n=15, 45%). Other significant categories included physical activity misinformation pertaining to general physical activity and messaging (n=6, 18%), exercising with a specific condition (n=4, 12%), women’s health (n=3, 9%), and weight loss (n=2, 6%). The remaining studies addressed misinformation concerning exercise testing (n=1, 3%), “immune boosting exercise” (n=1, 3%), and workplace sitting versus standing guidelines (n=1, 3%). Table 1 presents the characteristics of the included studies.

**Table 1:** Characteristics of included studies.

Study	Topic	Study design	Online media	Study population or content
Kanthawala et al [21], 2016	Specific condition: diabetes	Content analysis	WebMD and online searches	Online community responses versus search results (n=60 questions)
Michelini [22], 2017	General physical activity messaging	Content analysis	National health strategies	National physical activity messaging online (no participants)
Borah and Xiao [23], 2018	General physical activity messaging	Experiment	Facebook	Study 1 (physical activity): 340 US college students (female: 66.2%; mean age 19.8 y)
Chau et al [24], 2018	Workplace sitting guidelines	Content analysis	News articles	News articles (n=58)
Ekkekakis et al [25], 2018	Therapeutic exercise: depression	Internet search volume analysis and content analysis	Mass media and research articles	Research articles citing TREAD^a^ study (n=68)
Gonzalez [26], 2018	Weight loss	Pedagogical	Online media articles	College students (n=149)
Kocyigit et al [27], 2019	Rehabilitation: ankylosing spondylitis exercises	Content analysis	YouTube	Videos (n=56)
Dedrick et al [28], 2020	Weight loss: belly fat loss exercises	Content analysis	Pinterest	“Pins” (n=234)
Kunze et al [29], 2020	Rehabilitation: meniscus exercises	Content analysis	YouTube	Videos (n=50)
Rachul et al [30], 2020	Immune boosting	Content analysis	Google Search	Search result links (n=227)
Snyder et al [31], 2020	Women’s health: breastfeeding	Qualitative study using semistructured interviews	Facebook	Breastfeeding mothers (n=24)
Heisinger et al [32], 2021	Rehabilitation: lumbar disc herniation	Content analysis	YouTube	Videos (n=76)
Marocolo et al [33], 2021	General physical activity	Content analysis	Instagram	Posts (n=495)
Nagpal et al [34], 2021	Women’s health: HIIT^b^ during pregnancy	Content analysis	Google Search	Search result links (n=33)
Ori et al [35], 2021	General physical activity	Experiment	Blogs	Young women in Canada (n=141)
Yildiz and Toros [36], 2021	Rehabilitation: vertigo and vestibular disorders	Content analysis	YouTube	Videos (n=103)
Etzel et al [37], 2022	Rehabilitation: shoulder instability	Content analysis	YouTube	Videos (n=50)
Güloğlu et al [38], 2022	Rehabilitation: breast cancer surgery	Content analysis	YouTube	Videos (n=82)
Onder et al [39], 2022	Specific condition: osteoporosis	Content analysis	YouTube	Videos (n=238)
Rodriguez-Rodriguez et al [40], 2022	General physical activity	Content analysis	YouTube	Videos (n=68)
Yang et al [41], 2022	Rehabilitation: fall prevention	Content analysis	YouTube	Videos (workout subset n=58)
Yüce et al [42], 2022	Rehabilitation: patellofemoral instability	Content analysis	YouTube	Videos (n=89)
Zhang et al [43], 2022	Rehabilitation: neck pain	Content analysis	YouTube	Videos (n=20)
Anastasio et al [44], 2023	Rehabilitation: ankle sprain	Content analysis	TikTok	Videos (n=100)
Bethell et al [45], 2023	Rehabilitation: anterior cruciate ligament	Content analysis	TikTok	Videos (n=111)
O’Donnell et al [46], 2023	General physical activity	Content analysis	TikTok	Videos (n=400)
Tabarestani et al [47], 2023	Rehabilitation: Achilles tendinopathy	Content analysis	TikTok	Videos (n=100)
Nagasawa et al [48], 2024	Specific condition: hybrid assistive limb for neuromuscular disease or stroke	Content analysis	YouTube	Videos (n=100)
Rust et al [49], 2024	Rehabilitation: knee instability	Content analysis	TikTok	Videos (n=187)
Zure et al [50], 2024	Rehabilitation: fibromyalgia syndrome	Content analysis	YouTube	Videos (n=70)
Gong et al [51], 2025	Cardiopulmonary exercise testing	Content analysis	TikTok	Videos (n=48)
Pfender et al [52], 2025	Women’s health: cycle syncing	Content analysis	TikTok	Videos (n=100)
Rocha-Silva et al [53], 2025	Specific condition: epilepsy	Content analysis	AI^c^ chatbots	AI chatbots (n=4)

^a^TREAD: TREAtment of Depression with physical activity

^b^HIIT: high-intensity interval training.

^c^AI: artificial intelligence.

### Social Media Platform and Media Type

In the 33 studies, the most studied social media platform, YouTube (n=13, 40%), was used to evaluate the content quality and accuracy of 1060 videos. Similarly, TikTok was used in 7 (21%) studies to analyze the content of 1046 videos. Facebook (n=2, 6%) was the third most common platform included in the relevant studies, with 364 participants interacting with Facebook posts or describing their use of Facebook groups for information. Pinterest was used in one study, in which the content of 234 “pins” was analyzed for misinformation. The final social media platform included was Instagram (n=1, 3%), in which 495 posts from 33 prominent accounts (ie, influencers), with an average of more than 1 million followers, were evaluated. All other selected studies (n=9, 27%) used digital media that could be shared on social media, as opposed to content already shared on social media platforms. This included blogs, news articles, internet forums, or other online resources found from Google searches.

### Study Population and Design

Regarding the study populations and designs, four (12%) of the 33 studies involved data collection with human participants: (1) determining what college students (n=340) perceive as credible health information on Facebook [23]; (2) designing a module trial to train college students (n=149) on how to critically assess potentially misleading weight loss information online [26]; (3) assessing the believability of exercise blogs among young women (n=141) [35]; and (4) surveying breastfeeding women (n=24) for potential sources of misinformation, including Facebook groups [31]. The remaining 29 (88%) studies did not consist of actively recruited participants but analyzed readily available online content, including social media, news articles, websites, or blogs. Aside from observational study design (content analysis), the other study designs used were experimental, educational, and qualitative (using semistructured interviews).

### Prevalence, Reach, and Spread

We considered the measurement of misinformation in terms of prevalence (frequency among content), reach (how many individuals saw the content), and spread (whether the content was disseminated; Multimedia Appendix 2 [21-53]). A total of 27 (82%) of the 33 studies reported a measure of misinformation prevalence, the most common of which was video content quality, assessed using the Global Quality Score (GQS)—a tool to measure educational quality of online health-related content, with scores ranging from 1 (very poor quality) to 5 (excellent quality)—and DISCERN [54], an instrument for judging the quality of consumer health information on treatment choices, with scores ranging from 63 to 75 indicating excellent quality, 51 to 62 indicating good quality, 39 to 50 indicating fair quality, 27 to 38 indicating poor quality, and 16 to 26 indicating very poor quality, or 4 to 5 indicating high quality, 3 indicating moderate quality, and 1 to 2 indicating low quality for the modified DISCERN instrument [55].

Of the 33 studies, 12 (36%) studies using GQS for video content reported mean or median scores in the range of 2 (generally poor) to 3 (moderate quality), indicating a significant amount of missing information and high potential for misinformation across 818 videos analyzed. A total of 10 (30%) studies reported video content quality using the DISCERN or modified DISCERN instruments, with scores ranging from 25.9 (very poor) to 36.5 (fair) and 1 (low quality) to 3 (moderate quality), respectively, for 1080 videos.

Among 495 posts from the top Brazilian exercise and health Instagram accounts, Marocolo et al [33] found a high prevalence of relatively low-quality information, with an overall average quality rating of <39% and citations supporting the posted information in only 2.7% of the posts. In an analysis of 234 Pinterest pins, Dedrick et al [28] found significant missing information in 100% of the pins analyzed; 25.6% of the pins showed pictures that appeared to be touched up, and 11.5% showed before and after workout images that were not attainable based on the time frame. In 100 TikTok videos, Pfender et al [52] found that 57% of the creators recommended syncing the menstrual cycle to specific types of exercise, while the scientific literature lacks consensus on the topic [56].

A total of 3 (9%) of the 33 studies assessed Google Search results for misinformation prevalence, each finding high prevalence. In an analysis of guidance on HIIT for pregnant women, Nagpal et al [34] found that most of the linked resources lacked key information, such as adjusting exercise intensity for prepregnancy activity level (54.5%) or trimester (57.6%), and failed to mention contraindications to exercise (87.5%). Although it included more topics along with physical activity, a study comparing Google Search results with WebMD online community answers to diabetes-related health and wellness questions found that roughly half of the answers were clinically valid or accurate (50.8% and 52.5%, respectively) [21]. Rachul et al [30] found that exercise was recommended for “immune boosting” to prevent COVID-19 infection in 30.8% of the 227 search result webpages analyzed, which is not supported by science.

A total of 21 (64%) of the 33 studies measured the potential reach of physical activity misinformation, quantified as video views, account followers, channel subscribers, video “likes,” and article citations. In studies reporting video views, the range of median views per video was between 1168 and 375,039, and for those reporting mean views per video, the range was 16,471.5 to 288,597.7, with more than 43 million views. Account followers (Instagram) and channel subscribers (YouTube) were other metrics by which researchers reported the potential reach of misinformation. Marocolo et al [33] reported that the accounts analyzed in their study had a mean of 1,114,333 followers, while Zure et al [50] reported a mean of 745,476 channel subscribers in their study.

Studies measured the spread of misinformation in 2 ways. Ekkekakis et al [25] analyzed Google Search trends following a media campaign spreading misinterpreted scientific findings of a study regarding the effects of exercise on depression, finding a sharp increase of 357% in relevant searches in the month of the campaign compared to the subsequent 5 years. Several studies of TikTok were able to collect the median number of video shares, which ranged between 11 and 27 shares per video.

## Discussion

### Principal Findings

The purpose of this systematic review was to evaluate the state of the science related to physical activity misinformation on social media, including its reported prevalence, reach, and spread, and strategies for mitigating the issue. We found that no single physical activity topic dominated academic interest. Instead, a plurality of studies focused on physical rehabilitation and therapeutic exercise related to a wide variety of injuries or conditions. The topics related to physical activity misinformation that have been studied are as interesting as those that have received limited attention. Surprisingly, information about physical activity for weight loss was only targeted in 2 (6%) of the 33 studies. Given the public interest in weight loss, anecdotal evidence of misinformation on social media, and the history of pseudoscience and false claims, one might expect more scholarly attention to the subject. However, weight loss and similar broad topics may be challenging to study given the variety of potential misinformation.

Our review revealed that research into physical activity misinformation on social media is largely siloed, with most (24/33, 73%) studies focusing on a single social media platform. YouTube was the most frequently investigated platform, likely due to its vast repository of searchable video content and wide audience, with an estimated 81% of US adults having accessed YouTube at least once [57]. However, studies included in our review found the content to be of low-to-moderate quality, which is consistent with broader health topics on YouTube [58]. TikTok studies were also common and followed a similar methodology to those evaluating video content quality on YouTube; however, no study searched for misinformation across multiple social media platforms.

This focus on individual social media platforms fails to characterize the complex ecosystem in which misinformation spreads and may reflect methodological convenience. The narrow scope obscures critical aspects of the misinformation ecosystem, as Southwell et al [59] noted regarding the absence of misinformation research beyond social media data, potentially missing how misleading content moves between traditional media, interpersonal communication, and digital spaces. Therefore, while platform-specific analyses are useful, future research must also adopt broader approaches to better understand the cross-media dissemination of physical activity misinformation online. One approach could be to build on the method used by Ekkekakis et al [25], which involves examining the impact of a viral physical activity topic covered in mass media. Ekkekakis et al [25] found that following the publication of a particularly impactful research study, the relationship between depression and exercise was not accurately portrayed in the media. Moreover, it led to a surge in Google searches and was frequently misrepresented by other researchers who referred to the initial study. This could be extended further to evaluate the spread of this misinformation across social media platforms.

While content on a variety of social media platforms was investigated, our review identified that only 3 potentially overlapping populations have been recruited for research: college students, young women, and lactating women. This lack of diversity is a critical gap because, as Calac and Southwell [60] emphasize, misinformation exposure varies significantly across populations, yet current research fails to examine how factors such as socioeconomic status, race and ethnicity, geographic location, or disability status influence both exposure to and consequences of physical activity misinformation. This gap is especially problematic considering structural inequalities in access to safe physical activity environments where communities lacking secure outdoor spaces or well-equipped facilities may rely more heavily on online information that, if misleading, could exacerbate existing health disparities. Following the study by Southwell et al [61], future research must adopt an equity lens that examines the broader structural contexts shaping how different groups encounter and interpret health information, including those with varying levels of health literacy and cultural attitudes toward physical activity and institutional health messaging.

While studying physical activity misinformation on social media, researchers often aimed to quantify the prevalence, reach, and spread of misinformation. We found that 27 (82%) of the 33 studies reported a metric relating to the prevalence of misinformation on a physical activity topic. Methods of measuring misinformation prevalence included evaluating the accuracy of information in popular and high-ranked videos on YouTube and TikTok, top search results on Google Search, and messages from top influencers (ie, accounts with the most followers). This suggests that there are 2 approaches to evaluating misinformation online. Taking a search-driven approach, researchers can scrutinize individual pieces of content, whereas with an account-driven approach, researchers can examine accounts that have a significant number of followers and regularly disseminate misinformation to their audience.

Quantifying misinformation reach was reported in 21 (64%) of the 33 studies. The metrics used to gauge reach included video views, account followers, channel subscribers, video “likes,” and article citations. We found the median views reported were from 4100 to 375,000 per video, with total views as high as 7.7 million, which indicates a wide reach, although it is not as wide as some health topics, such as the COVID-19 vaccine [62]. Furthermore, the range of views elucidates the need for misinformation prevalence and reach to be examined together. For example, Güloğlu et al [38] showed that while misleading videos were prevalent (51.2%) and of a significantly lower quality (GQS=2 vs. GQS=4), they had a lower reach compared with videos classified as useful—25.7% and 74.3% of total views, respectively. However, in other cases, the opposite can easily be true, as studies noted that content popularity had no correlation or negative correlation with content quality [32,39,43,49].

While knowing the reach of misinformation is important for understanding the dynamics of misinformation, measuring its spread is equally critical, yet this metric was not often reported in the included studies. Of the 33 studies, our review identified 1 (3%) study that quantified the spread of physical activity misinformation through Google Search trends and several (n=5, 15%) others through the number of video shares on TikTok. More work needs to be done to fully understand how physical activity misinformation propagates within and across various platforms.

### Recommendations for Combating Misinformation

Our review of the current literature suggests that the challenge of physical activity misinformation on social media is unlikely to be resolved without proactive intervention. On the basis of the evidence, a multipronged approach is necessary to mitigate its impact. First, the quality and framing of physical activity messaging are critical. Content should be framed positively, highlighting short-term social and mental health benefits, and should be tailored to the intended audience using formative research and established psychological principles [63]. Content seeking to debunk misinformation should target specific misconceptions [64] and information seekers with a personal connection to the issue [13]. This approach of creating high-quality, targeted content should be adopted by institutions to directly compete with the deluge of poor-quality information online. With that goal, Haslam et al [65] have described 10 factors to enhance the accessibility of credible health content on platforms such as YouTube, which is essential for reaching a wide audience.

In addition to these social media content strategies, health professionals can play a vital preventive role during patient consultations by ensuring patients’ questions are fully addressed, as unresolved queries often lead individuals to seek information from less reliable online sources [31]. Finally, the spread of misinformation can also be disrupted at a key source: the communication of health research to the public. Academic journals writing press releases for studies should be mindful that news organizations often lack the incentive or expertise to highlight flaws in research methods and are not inclined to revisit a nuanced topic when a similar study is later published [66]. By implementing strategies that focus on creating high-quality content, improving professional practice, and ensuring accurate science communication, the impact of health misinformation can be reduced.

### Areas for Future Research

While this review describes the current state of research on physical activity misinformation on social media, it also reveals gaps that future research must address. First, there is a lack of research extending beyond content analysis to measure the impact of physical activity misinformation on real-world behavior. Future research should use experimental designs to test how exposure to specific types of physical activity misinformation influences intentions, decisions, and subsequent behaviors. Second, the current research landscape is predominantly cross-sectional “snapshots” of misinformation on single platforms, which fails to capture how misleading narratives evolve and spread. Therefore, we recommend longitudinal studies to investigate the misinformation over time. Third, manual analysis methods (eg, GQS and DISCERN) common among the studies we reviewed are not scalable for comprehensive, real-time detection. A promising area for future work is the development and validation of artificial intelligence–driven tools to detect physical activity misinformation. These models could be engineered to identify unsubstantiated claims (eg, “immune boosting” exercise) and pseudoscientific language specific to fitness, health, and wellness. Large language models have shown effectiveness in this area [67,68], but caution should be taken as large language models have also been shown to propagate health misinformation [69,70]. Addressing these areas will move the field beyond describing the problem of physical activity misinformation on social media toward mitigating its impact on public health.

### Limitations

Our systematic review of physical activity misinformation on social media has several limitations that readers and future researchers in the field should consider. In many (10/33, 30%) of the articles that met our inclusion criteria, the words misinformation, fake news, or disinformation were not used. Instead, these articles discussed the accuracy, quality, or validity of content. While we used a wide array of search terms, there may have been similar keywords we missed, which leaves the possibility that our search excluded relevant articles. In our search, there was also potential bias introduced by the selected databases and self-reported data in some studies, along with publication bias, which means studies finding minimal misinformation may be underrepresented in the published literature. We omitted any articles not published in English. As social media, physical activity, and misinformation are not limited to the English-speaking world, this may have excluded relevant articles. In our review, we found high variability in study topics, designs, and purposes and limited coverage of topics, platforms, and populations, making it difficult to conduct a traditional quality assessment or draw firm conclusions. Often, studies did not share the same outcome measures or report the same metrics related to misinformation, limiting synthesis and the ability to apply a misinformation classification scheme. Finally, we found a lack of longitudinal studies tracking misinformation over time, which limits our ability to draw conclusions about its impact.

### Conclusions

The objective of this systematic review was to identify original research studies on physical activity misinformation on social media to better understand its prevalence, reach, and spread. In addition, we aimed to highlight ways to mitigate its impact and areas for further investigation. Our review revealed that physical activity misinformation is a multifaceted issue that presents in various forms across a range of physical activity topics, social media platforms and types, study populations, and study designs. Therefore, studying this issue requires a multidimensional approach that uses a diversity of research methods. Our hope is that future researchers recognize this complexity and explore new avenues for investigating the dissemination and propagation of misinformation, especially across social media platforms.

## Appendix

Multimedia Appendix 1 PRISMA checklist.

Multimedia Appendix 2 Measures of misinformation prevalence, reach, and spread.

## Abbreviations

GQS: Global Quality Score
HIIT: high-intensity interval training
MeSH: Medical Subject Headings
PRISMA: Preferred Reporting Items for Systematic Reviews and Meta-Analyses
