# Supplementary material for: Physical Activity Misinformation on Social Media: Systematic Review
Source: JMIR Infodemiology. 2025 Oct 8;5:e62760. doi: 10.2196/62760 (PMC12547344; doi:10.2196/62760)
Supplement: Multimedia Appendix 2 [file infodemiology_v5i1e62760_app2.docx]

| Authors | Prevalence measure of misinformation | | Reach measure of misinformation | | Spread measure of misinformation |
| --- | --- | --- | --- | --- | --- |
| Kanthawala et al. [21], 2016 | Clinical relevance & validity | - | | - | |
| Michelini [22], 2017 | - | - | | - | |
| Borah & Xiao [23], 2018 | - | - | | - | |
| Chau et al. [24], 2018 | Article content | - | | - | |
| Ekkekakis et al. [25], 2018 | Article content accuracy | Citations | | Google Search trends | |
| Gonzalez [26], 2018 | - | - | | - | |
| Kocyigit et al. [27], 2019 | Video content quality | Video views | | - | |
| Dedrick et al. [28], 2020 | “Pin” content | Followers of “Pin” creators | | - | |
| Kunze et al. [29], 2020 | Video content quality | Video views | | - | |
| Rachul et al. [30], 2020 | Webpage content | - | | - | |
| Snyder et al. [31], 2020 | - | - | | - | |
| Heisinger et al. [32], 2021 | Video content quality | Video views | | - | |
| Marocolo et al. [33], 2021 | Post content | Account followers | | - | |
| Nagpal et al. [34], 2021 | Webpage content quality | - | | - | |
| Ori et al. [35], 2021 | - | - | | - | |
| Yildiz & Toros [36], 2021 | Video content quality | Video views | | - | |
| Etzel et al. [37], 2022 | Video content quality | Video views | | - | |
| Güloğlu et al. [38], 2022 | Video content quality | Video views | | - | |
| Onder et al. [39], 2022 | Video content quality | Video views | | - | |
| Rodriguez-Rodriguez et al. [40], 2022 | Video content quality | Video views | | - | |
| Yang et al. [41], 2022 | Video content quality | Video views | | - | |
| Yüce et al. [42], 2022 | Video content quality | Video views | | - | |
| Zhang et al. [43], 2022 | Video content quality | Video views | | - | |
| Anastasio et al. [44], 2023 | Video content quality | Video views | | Video shares | |
| Bethell et al. [45], 2023 | Video content quality | Video views | | Video shares | |
| O’Donnell et al. [46], 2023 | - | - | | - | |
| Tabarestani et al. [47], 2023 | Video content quality | Video views | | Video shares | |
| Nagasawa et al. [48], 2024 | Video content quality | Video views | | - | |
| Rust et al. [49], 2024 | Video content quality | Video views | | Video shares | |
| Zure et al. [50], 2024 | Video content quality | Channel subscribers | | - | |
| Gong et al. [51], 2025 | Video content quality | - | | Video shares | |
| Pfender et al. [52], 2025 | Video content | Video “likes” | | - | |
| Rocha-Silva et al. [53], 2025 | AI chatbot response quality | - | | - | |

### References

21. Kanthawala S, Vermeesch A, Given B, Huh J. Answers to Health Questions: Internet Search Results Versus Online Health Community Responses. *J Med Internet Res*. 2016;18(4):e95. doi:10.2196/jmir.5369

22. Michelini E. Communicative validity of health-related promotion of physical activity: A critical analysis of national health strategies. *SOCIAL THEORY & HEALTH*. 2017;15(4):448-464. doi:10.1057/s41285-017-0037-2

23. Borah P, Xiao X. The Importance of ‘Likes’: The Interplay of Message Framing, Source, and Social Endorsement on Credibility Perceptions of Health Information on Facebook. *Journal of Health Communication*. 2018;23(4):399-411. doi:10.1080/10810730.2018.1455770

24. Chau JY, McGill B, Freeman B, Bonfiglioli C, Bauman A. Overselling Sit-Stand Desks: News Coverage of Workplace Sitting Guidelines. *Health Communication*. 2018;33(12):1475-1481. doi:10.1080/10410236.2017.1359034

25. Ekkekakis P, Hartman ME, Ladwig MA. Mass media representations of the evidence as a possible deterrent to recommending exercise for the treatment of depression: Lessons five years after the extraordinary case of TREAD-UK. *Journal of Sports Sciences*. 2018;36(16):1860-1871. doi:10.1080/02640414.2018.1423856

26. Gonzalez JT. Using misleading online media articles to teach critical assessment of scientific findings about weight loss. *Advances in Physiology Education*. 2018;42(3):500-506. doi:10.1152/advan.00065.2018

27. Kocyigit BF, Nacitarhan V, Koca TT, Berk E. YouTube as a source of patient information for ankylosing spondylitis exercises. *Clin Rheumatol*. 2019;38(6):1747-1751. doi:10.1007/s10067-018-04413-0

28. Dedrick A, Merten J, Adams T, Wheeler M, Kassie T, King J. A Content Analysis of Pinterest Belly Fat Loss Exercises: Unrealistic Expectations and Misinformation. *AMERICAN JOURNAL OF HEALTH EDUCATION*. 2020;51(5):328-337. doi:10.1080/19325037.2020.1795754

29. Kunze KN, Krivicich LM, Verma NN, Chahla J. Quality of Online Video Resources Concerning Patient Education for the Meniscus: A YouTube-Based Quality-Control Study. *Arthroscopy*. 2020;36(1):233-238. doi:10.1016/j.arthro.2019.07.033

30. Rachul C, Marcon A, Collins B, Caulfield T. COVID-19 and “immune boosting” on the internet: a content analysis of Google search results. *BMJ OPEN*. 2020;10(10). doi:10.1136/bmjopen-2020-040989

31. Snyder K, Pelster AK, Dinkel D. Healthy eating and physical activity among breastfeeding women: the role of misinformation. *BMC Pregnancy Childbirth*. 2020;20(1):470. doi:10.1186/s12884-020-03153-x

32. Heisinger S, Huber D, Matzner MP, et al. Online Videos as a Source of Physiotherapy Exercise Tutorials for Patients with Lumbar Disc Herniation-A Quality Assessment. *Int J Environ Res Public Health*. 2021;18(11). doi:10.3390/ijerph18115815

33. Marocolo M, Meireles A, de Souza HLR, et al. Is Social Media Spreading Misinformation on Exercise and Health in Brazil? *Int J Environ Res Public Health*. 2021;18(22). doi:10.3390/ijerph182211914

34. Nagpal TS, Everest C, Goudreau AD, Manicks M, Adamo KB. To HIIT or not to HIIT? The question pregnant women may be searching for online: a descriptive observational study. *Perspect Public Health*. 2021;141(2):81-88. doi:10.1177/1757913920985898

35. Ori EM, Berry TR, Yun L. The Believability of Exercise Blogs Among Young Adults. *J Sport Exerc Psychol*. 2021;43(1):53-60. doi:10.1123/jsep.2020-0177

36. Yildiz S, Toros SZ. The Quality, Reliability, and Popularity of YouTube Education Videos for Vestibular Rehabilitation: A Cross-sectional Study. *Otol Neurotol*. 2021;42(8):e1077-e1083. doi:10.1097/MAO.0000000000003197

37. Etzel CM, Bokshan SL, Forster TA, Owens BD. A quality assessment of YouTube content on shoulder instability. *Phys Sportsmed*. 2022;50(4):289-294. doi:10.1080/00913847.2021.1942286

38. Güloğlu S, Özdemir Y, Basim P, Tolu S. YouTube English videos as a source of information on arm and shoulder exercise after breast cancer surgery. *European Journal of Cancer Care*. 2022;31(6):e13685. doi:10.1111/ecc.13685

39. Onder ME, Onder CE, Zengin O. Quality of English-language videos available on YouTube as a source of information on osteoporosis. *Arch Osteoporos*. 2022;17(1):19. doi:10.1007/s11657-022-01064-2

40. Rodriguez-Rodriguez AM, Blanco-Diaz M, de la Fuente-Costa M, Hernandez-Sanchez S, Escobio-Prieto I, Casaña J. Review of the Quality of YouTube Videos Recommending Exercises for the COVID-19 Lockdown. *Int J Environ Res Public Health*. 2022;19(13):8016. doi:10.3390/ijerph19138016

41. Yang X, Xue X, Shi Z, et al. The reliability, functional quality, understandability, and actionability of fall prevention content in YouTube: an observational study. *BMC Geriatr*. 2022;22(1):654. doi:10.1186/s12877-022-03330-x

42. Yüce A, İğde N, Ergün T, Mısır A. YouTube provides insufficient information on patellofemoral instability. *Acta Orthop Traumatol Turc*. 2022;56(5):306-310. doi:10.5152/j.aott.2022.22005

43. Zhang X, Yang Y, Shen YW, et al. Quality of online video resources concerning patient education for neck pain: A YouTube-based quality-control study. *Front Public Health*. 2022;10:972348. doi:10.3389/fpubh.2022.972348

44. Anastasio AT, Tabarestani TQ, Bagheri K, et al. A New Trend in Social Media and Medicine: The Poor Quality of Videos Related to Ankle Sprain Exercises on TikTok. *Foot & Ankle Orthopaedics*. 2023;8(2):24730114231171117. doi:10.1177/24730114231171117

45. Bethell MA, Anastasio AT, Adu-Kwarteng K, Tabarestani TQ, Lau BC. Analyzing the Quality, Reliability, and Educational Value of ACL Rehabilitation Exercises on TikTok: A Cross-Sectional Study. *Orthopaedic Journal of Sports Medicine*. 2023;11(12):23259671231218668. doi:10.1177/23259671231218668

46. O’Donnell N, Jerin SI, Mu D. Using TikTok to Educate, Influence, or Inspire? A Content Analysis of Health-Related EduTok Videos. *Journal of Health Communication*. 2023;28(8):539-551. doi:10.1080/10810730.2023.2234866

47. Tabarestani TQ, Anastasio AT, Duruewuru A, Taylor J, Bethell MA, Adams S. Analyzing the Quality and Educational Value of Achilles Tendinopathy-Related Videos on TikTok. *Foot & Ankle Orthopaedics*. 2023;8(4):2473011423S00230. doi:10.1177/2473011423S00230

48. Nagasawa M, Nakamura S, Narimatsu H. Characteristics of the most viewed Hybrid Assistive Limb-related videos on YouTube. *Health Informatics J*. 2024;30(1):14604582241236697. doi:10.1177/14604582241236697

49. Rust B, Christoforides E, Singh A, et al. Evaluating the Distribution, Quality, and Educational Value of Videos Related to Knee Instability Exercises on the Social Media Platform TikTok. *CUREUS JOURNAL OF MEDICAL SCIENCE*. 2024;16(3). doi:10.7759/cureus.57104

50. Zure M, Korkmaz M, Menekseoglu A. Exercises for fibromyalgia syndrome: what YouTube tells us as a source of information for patient and physician education. *CLINICAL RHEUMATOLOGY*. 2024;43(1):473-480. doi:10.1007/s10067-023-06792-5

51. Gong X, Zhang Z, Dong B, Pan H. TikTok’s cardiopulmonary exercise testing videos: A content analysis of quality and misinformation. *DIGITAL HEALTH*. 2025;11. doi:10.1177/20552076251341090

52. Pfender E, Wanzer C, Mikkers L, Bleakley A. Sync or Swim: Navigating the Tides of Menstrual Cycle Messaging on TikTok. *Perspect Sex Reprod Health*. Published online March 17, 2025. doi:10.1111/psrh.70004

53. Rocha-Silva R, de Lima BE, Costa TG, et al. Can people with epilepsy trust AI chatbots for information on physical exercise? *Epilepsy Behav*. 2025;163:110193. doi:10.1016/j.yebeh.2024.110193
